# Application of affymetrix array and massively parallel signature sequencing for identification of genes involved in prostate cancer progression

**DOI:** 10.1186/1471-2407-5-86

**Published:** 2005-07-22

**Authors:** Asa J Oudes, Jared C Roach, Laura S Walashek, Lillian J Eichner, Lawrence D True, Robert L Vessella, Alvin Y Liu

**Affiliations:** 1 Institute for Systems Biology, Seattle, USA; 2 Department of Urology, University of Washington, Seattle, USA; 3 Department of Pathology, University of Washington, Seattle, USA

## Abstract

**Background:**

Affymetrix GeneChip Array and Massively Parallel Signature Sequencing (MPSS) are two high throughput methodologies used to profile transcriptomes. Each method has certain strengths and weaknesses; however, no comparison has been made between the data derived from Affymetrix arrays and MPSS. In this study, two lineage-related prostate cancer cell lines, LNCaP and C4-2, were used for transcriptome analysis with the aim of identifying genes associated with prostate cancer progression.

**Methods:**

Affymetrix GeneChip array and MPSS analyses were performed. Data was analyzed with GeneSpring 6.2 and in-house perl scripts. Expression array results were verified with RT-PCR.

**Results:**

Comparison of the data revealed that both technologies detected genes the other did not. In LNCaP, 3,180 genes were only detected by Affymetrix and 1,169 genes were only detected by MPSS. Similarly, in C4-2, 4,121 genes were only detected by Affymetrix and 1,014 genes were only detected by MPSS. Analysis of the combined transcriptomes identified 66 genes unique to LNCaP cells and 33 genes unique to C4-2 cells. Expression analysis of these genes in prostate cancer specimens showed CA1 to be highly expressed in bone metastasis but not expressed in primary tumor and EPHA7 to be expressed in normal prostate and primary tumor but not bone metastasis.

**Conclusion:**

Our data indicates that transcriptome profiling with a single methodology will not fully assess the expression of all genes in a cell line. A combination of transcription profiling technologies such as DNA array and MPSS provides a more robust means to assess the expression profile of an RNA sample. Finally, genes that were differentially expressed in cell lines were also differentially expressed in primary prostate cancer and its metastases.

## Background

Profiling the expression pattern of genes in a tissue or cultured cells is often a starting point for exploratory genomic studies. Serial analysis of gene expression (SAGE) [1] is a technology for gene expression studies that can provide whole transcriptome coverage; however, it is slow and relatively labor intensive because each clone that is generated during library construction must be sequenced. The invention of DNA microarray technology [2,3], in combination with sequence information for the human genome [4,5] has provided the ability to rapidly assess the transcriptome profile of an RNA sample. A recently developed technology called massively parallel signature sequencing (MPSS) [6] allows the transcriptome of an RNA sample to be determined without prior genomic knowledge of the organism under study. An advantage that MPSS has over DNA microarrays is that expression of unknown genes can be observed since no sequence specific nucleic acid probes are required for detection. MPSS is akin to SAGE in that it uses short sequence tags to identify transcripts, however, the techniques differ in the method used for sequence determination. In MPSS, sequencing of the entire library occurs in parallel on microbeads by multiple rounds of enzymatic cleavage followed by ligation of labeled adaptors to identify the sequence revealed by the enzymatic cleavage; as opposed to chain termination DNA sequencing of individual clones in SAGE. MPSS has been used to profile the gene expression of animal cell lines [7], *Arabidopsis *[8,9], and maize [10].

Prostate cancer is the most common cancer diagnosed in American males and the second leading cause of cancer death in men [11,12]. Primary prostate cancer typically requires androgen to maintain its growth. Consequently, the growth and clinical effects of progressive prostate cancer characteristically respond to androgen ablation therapy. Although androgen ablation initially retards the growth of metastatic prostate cancer, ultimately, the disease escapes androgen blockade and evolves to an androgen independent state [13,14]. The later stages of progressive prostate cancer are characterized by a high frequency of bone metastasis [15]. Since androgen independent prostate cancer causes significant morbidity, ultimately leading to mortality, and since there is no generally effective therapy for this state of prostate cancer, characterizing differences between androgen-responsive and androgen-independent prostate cancer is of great importance, and may reveal mechanisms or targets for molecular therapy. In this study, we compare the ability of Affymetrix GeneChip DNA microarrays and MPSS to determine gene expression profiles of two prostate cancer cell lines. We determined the transcription profile of LNCaP [16,17] and LNCaP derived C4-2 cells [18], with the aim of identifying genes involved in the progression of prostate cancer as modeled by the transition of LNCaP to C4-2 and their expression pattern in prostate cancer.

## Methods

### Cultured cells and RNA preparation

LNCaP and C4-2 cells were cultured in RPMI 1640 media supplemented with 5% fetal calf serum. Cells were harvested when 70–80% confluent. RNA was prepared from 10^7 ^cells using an RNeasy Mini Total RNA kit (Qiagen, Valencia, CA). RNA purity was assessed by UV absorbance and its quality with an Agilent 2100 Bioanalyzer and RNA 6000 Nano Labchips (Agilent, Foster City, CA).

### Prostate tissues

All tissue samples were obtained under the University of Washington Institutional Review Board protocol number: 00-3449-A03, based upon the following methods. Normal prostate parenchyma and primary prostate cancer tissue samples were collected from radical prostatectomy specimens following a standard procedure. To minimize RNA degradation, upon receipt of the radical prostatectomy specimen 3 mm thick transverse sections were made after inking the exterior surface (the surgical margin). Tissue blocks from the posterior aspect of each alternate transverse section were embedded in Tissue-Tek OCT (Sakura Finetek, Torrance, CA) and snap frozen in isopentane that had been pre-cooled in liquid nitrogen. Frozen sections of these blocks provided a template for identifying the portion of the blocks that contained cancer. A small fragment of cancer-enriched tissue (approximately 2 × 1 × 1 mm) was excised. Lymph node and bone metastasis specimens were collected from Rapid Autopsy of donor patients [19]. All tissue samples were placed in RNA Later (Ambion, Austin, TX) and stored at -20C prior to processing. RNA was prepared from these samples using STAT-60 (Tel-Test, Friendswood, TX). Briefly, 50–100 mg of tissue were placed in 1 ml STAT-60 and homogenized with an OmniTH using Omni Tip disposable generator probes (Omni, Marietta, GA). RNA was extracted with the addition of 0.2 ml chloroform. After centrifugation, the aqueous phase was centrifuged with 0.5 ml isopropanol. The RNA was resuspended in THE RNA solution (Ambion, Austin, TX). RNA purity and quality were assessed by absorbance and agarose gel electrophoresis.

### Affymetrix Arrays

Gene expression in LNCaP and C4-2 cells was analyzed with Human Genome U133 Plus 2.0 GeneChips (Affymetrix, Santa Clara, CA). Two individual biological replicate samples of LNCaP and C4-2 RNA were assayed. GeneChips were prepared, hybridized, and scanned according to the manufacturer's instruction. Briefly, 1 μg total RNA was reverse transcribed with a poly-(T) primer containing a T7 promoter, and the cDNA made double-stranded. An *in vitro *transcription was done to produce biotinylated cRNA, which was then hybridized to the GeneChips. The chips were washed and stained with streptavidin phycoerythrin using an Affymetrix FS-450 fluidics station, and data was collected with Affymetrix GeneChip Scanner 3000.

### MPSS

One sample of total RNA from C4-2 and LNCaP cell lines were submitted to Lynx Therapeutics (Hayward, CA) for MPSS analysis. The samples were processed and analyzed as described by Brenner *et al*. [6,20]. Briefly, mRNA was reverse transcribed and the cDNA was digested with the restriction enzyme *Dpn*II. The cDNA fragment between the poly-(A) tail and the *Dpn*II site was cloned. The resultant library was amplified and conjugated to microbeads. The microbeads were loaded into flow cells and the signature sequences were determined by a series of enzymatic reactions. The abundance for each signature in the pool was represented as transcripts per million (tpm).

### Gene expression analysis by RT-PCR

Reverse transcriptase-polymerase chain reaction (RT-PCR) was used to validate expression. For the cell lines, 1 μg RNA was reverse transcribed with superscript II reverse transcriptase (Invitrogen, Carlsbad, CA) at 50C for 50 min followed by 10 min at 70C. For tissue specimens, 1 μg RNA was reverse transcribed with Clontech Advantage RT (Clontech, Palo Alto, CA) at 42C for 1 h followed by 5 min at 94C. Primers for PCR [see Additional file 1] were designed to produce amplicons from 100–300 bp. PCR was carried out at 95C 30 s, 55C 30 s, 72C 1 min for 35 cycles. PCR products were resolved on 2% agarose gels.

### Data analysis

Affymetrix data was analyzed with GCOS version 1.0 software package (Affymetrix). Scanned images of the arrays were converted to numerical data by GCOS and outputted to tab delimited text files containing Affymetrix ProbeSet ID, signals, present or absent detection calls, and detection P-values for each feature on the array. The data was imported into GeneSpring 6.2 (Silicon Genetics, Redwood City, CA) and analyzed to determine expression profiles. The raw data was filtered to mask genes with signal intensities less than 50, which is at the background threshold, and to retain only genes that were called present by GCOS in both replicates. In order to map Affymetrix ProbeSet IDs to NCBI GeneIDs (previously Locuslink IDs) the Affymetrix ProbeSet annotation file "HG-U133_Plus_2_annot.csv" was parsed by perl scripts available at the Institute for Systems Biology (ISB) website [21]. If a GeneID was present in the "LocusLink" column, that GeneID was used for mapping. If not, the sequences in the columns "SwissProt" and "RefSeq Transcript ID" were queried against the mapping provided by Locuslink or its successor EntrezGene. If one GeneID could be mapped, it was used. Information from Affymetrix's "Target Description" column was not used. GeneIDs with no mapping to the Affymetrix ProbeSet were considered to be unrepresented in the Affymetrix dataset. Affymetrix ProbeSet annotation files are downloadable and are updated regularly. For this work, an annotation file downloaded in the second quarter of 2004 was used. The Affymetrix data was visualized with Mathematica version 5.0 (Wolfram Research, Champaign, IL).

MPSS data was received from Lynx Therapeutics in the form of tab delimited text files that listed signature DNA sequences and the bead counts for individual sequences. Many signatures were manually mapped using a graphical web interface provided by Lynx: the Signome browser. Other non-repetitive signatures were mapped with perl scripts by combining 3'-most signatures of all high-quality sequences known to correspond to a GeneID. Sequences acquired directly from Locuslink or EntrezGene were considered to be of high quality. Sequences from UniGene were considered to be of high quality only if they were of "complete cds" Refseqs or had both a predicted polyadenylation signal and a polyadenylated end. The counts of all signatures mapping to a GeneID were summed to get the tpm for that GeneID, for most genes the majority of counts were contributed by a single signature. GeneIDs with no signatures were considered to be unrepresented in the MPSS dataset. The perl scripts used to map MPSS tags to GeneIDs are also available at the ISB website [21]. The processed MPSS data was imported into GeneSpring in the form of a tab delimited text file containing GeneID and tpm counts associated with the genes. The datasets were filtered to retain genes with signal of ≥ 1 tpm, and the resulting lists of genes were considered to be the transcriptomes of the cell lines. MPSS data was also visualized with Mathematica.

For direct comparison of Affymetrix data to MPSS data we used only GeneIDs with at least one MPSS signature and did not consider ProbeSets with an "X" suffix. For a GeneID to be considered present in Affymetrix data, at least one ProbeSet assigned to that GeneID had to have a "present" call. For a GeneID to be considered absent, all ProbeSets assigned to that GeneID had to have an "absent" call. Each GeneID may be represented up to four times, for the two replicate Gene Chips analyzed for each of the two samples. A present call increases the odds that a transcript is expressed at a higher level, but does not rule out the possibility of zero expression. Likewise, an absent call increases the probability that expression is truly zero, but does not rule out the possibility of significant expression.

## Results

### Overall expression profiles

In the Affymetrix datasets, a low stringency evaluation of the transcriptome was determined by selecting genes that were detected at a raw fluorescence signal intensity of ≥ 50, which is higher than the average background signal level of 35 ± 3 for the LNCaP chips and 43 ± 7 for the C4-2 chips. The data was also parsed to select genes called present in both replicates of each cell line. We chose the cutoff points of ≥ 50 raw-signal and a call of present in both replicates for Affymetrix data because it was the most liberal method of determining if a gene was expressed by a cell-line. In establishing the cutoff points it was our aim to limit bias in our initial data analysis so that further analyses would have the maximum potential data to work with. Gene lists produced by the initial low stringency analysis were considered to be the transcriptome of each respective cell line. We define "gene" as a National Center for Biotechnology Information (NCBI) GeneID with corresponding expression level information from our dataset. Together, the LNCaP and C4-2 cells expressed 10,308 genes. Individually, LNCaP cells expressed 9,841 genes and C4-2 cells expressed 9,653 genes. Comparison of the genes expressed by LNCaP and C4-2 cells revealed that 9,186 of the genes were expressed in common, 655 genes were unique to LNCaP and 467 genes were unique to C4-2. To further refine the data for unique genes, select genes were culled from each cell-type specific dataset. For example, in the LNCaP dataset of unique genes all genes with raw signals <100 in LNCaP and >100 in C4-2 were removed. Next, all genes with GCOS software calls of absent in LNCaP and present in C4-2 were removed. The final number of genes unique to LNCaP was 172. The same filtering process was applied to the C4-2 dataset to produce a final list of 149 genes. Affymetrix data for LNCaP and C4-2 cell lines including .CHP files can be obtained from our website [22].

MPSS data was pared down to a gene list for each dataset that contained a GeneID number and the tpm for that gene. Similar to the initial analysis of Affymetrix data, simplified MPSS gene lists were evaluated at low stringency to determine the transcriptome of LNCaP and C4-2 cell lines. All genes present at ≥ 1 tpm in either one of the two datasets were enumerated. Like the Affymetrix data, the ≥ 1 tpm cutoff was chosen because it was the most liberal definition of gene expression for the MPSS data. Together, the LNCaP and C4-2 cells expressed 8,572 genes. Individually, LNCaP expressed 7,863 genes and C4-2 cells expressed 6,539 genes. Comparison of the low stringency gene lists revealed that LNCaP expressed 2,033 unique genes and C4-2 expressed 709 unique genes. To further refine the data, genes with a tpm of >3 in either sample were retained and genes with a tpm of <3 in both samples were eliminated from the overall expression profile. A tpm of 3 approximately corresponds to one transcript per cell [7] and is near the reliable detection limit of the current MPSS protocol. The refined data contained 5,806 genes common to the cell lines, 1,797 genes unique to LNCaP, and 658 genes unique to C4-2. MPSS data for LNCaP and C4-2 cell lines can be obtained from our website [22].

### Correlation between Affymetrix and MPSS data

To determine the degree to which the Affymetrix and MPSS data correlate, the transcriptomes assessed by each technique were compared by NCBI GeneID with Matchminer build 127 [23] merge algorithm and in-house perl scripts. Data from Matchminer and perl scripts were similar, and we present the data from our in-house analysis. Raw, low stringency selection data for both cell lines contained 10,308 and 8,586 GeneIDs for the Affymetrix and MPSS datasets respectively. Merging the datasets identified 3,050 genes detected by Affymetrix but not detected by MPSS, 1,328 genes detected by MPSS but not detected by Affymetrix, and 7,258 genes detected by both. In all, a combined transcriptome of 11,636 genes was established for LNCaP and C4-2.

**Figure 1:**
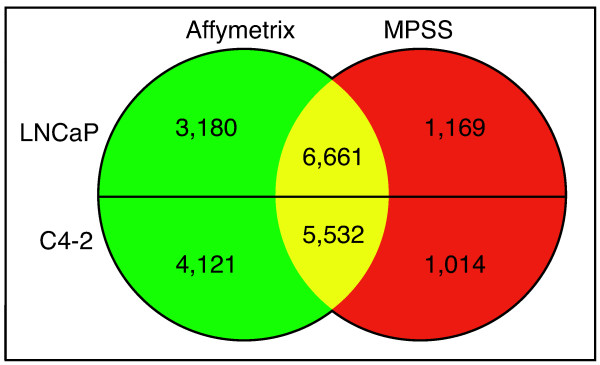
**Merged Affymetrix and MPSS transcriptome profiles. **Number of genes detected by Affymetrix and MPSS expression profiling of LNCaP (top) and C4-2 (bottom) cell lines. The number of genes detected by both platforms is listed in the yellow area, genes unique to Affymetrix are in the green area, and genes unique to MPSS are in the red area. Thousands of genes were only detected by one technology in each respective cell line.

We next directly compared the cell type-specific data produced by Affymetrix and MPSS (Fig. 1). For LNCaP, the Affymetrix dataset contained 9,841 genes, while the MPSS dataset contained 7,863 genes. Merging the LNCaP datasets showed that 3,180 genes were unique by Affymetrix, 1,169 genes were unique by MPSS, and 6,661 genes were detected by both technologies. For C4-2, the Affymetrix dataset contained 9,653 genes, while the MPSS dataset contained 6,539 genes. Merging the C4-2 datasets showed that 4,121 genes were unique by Affymetrix, 1,014 genes were unique by MPSS, and 5,532 genes were detected by both technologies [see Additional file 2].

### Raw signal comparison

**Figure 2:**
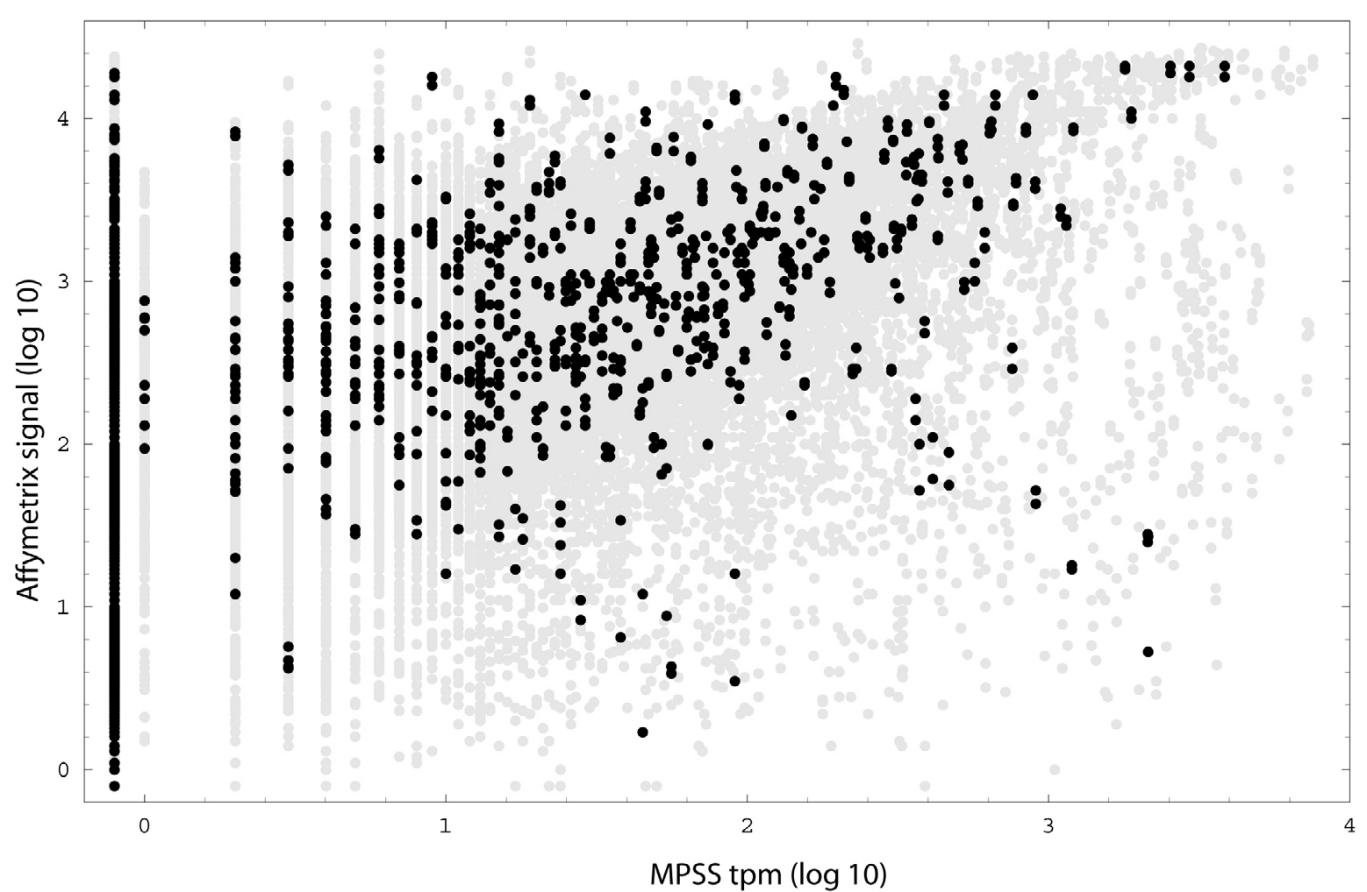
**Signal comparison of Affymetrix and MPSS results. **GeneID with manually curated signature mappings are in black, and those with automated mappings are in gray. All values are in log_10_, except zeros, which are portrayed as -0.1. Each GeneID is represented four times (for the two replicate GeneChips analyzed for each sample). A general trend of a monotonic increase with Affymetrix signal saturation at high expression is observed. However, variability is too great to justify a specific curve fit.

If the Affymetrix signal is highly correlated to the amount of transcript, it can be used to directly estimate the probability of the presence or absence of a particular transcript. To evaluate this correlation, we compared the Affymetrix signal to the MPSS tpm for each possible GeneID (Fig. 2). A number of factors could affect the implementation of this approach: (1) the Affymetrix signal is influenced by the distance of the ProbeSet from the 3' end of the transcript, (2) the Affymetrix signal is influenced by sequences of the probe pairs in a ProbeSet, (3) a ProbeSet assigned to a GeneID may be measuring a set of splice variants that is different from the set of splice variants measured by MPSS, and (4) gene models are imperfect in that the bioinformatics assigning ProbeSets and signatures to GeneIDs is prone to error. Furthermore (5), measurement of outliers for either or both technologies can randomly obscure the correlation between tpm and Affymetrix signal. To minimize these influences, we: (1) used only GeneIDs with at least one MPSS signature, (2) did not consider ProbeSets with an "X" suffix, (3) used the ProbeSet with the strongest signal for that condition if more than one ProbeSet was assigned to a GeneID, and (4) used established statistical methods [24,25] to limit spurious data contributed by outliers. Note that Affymetrix does not endorse the use of signal to predict transcript level.

### MPSS tpm and Affymetrix detection calls

**Figure 3:**
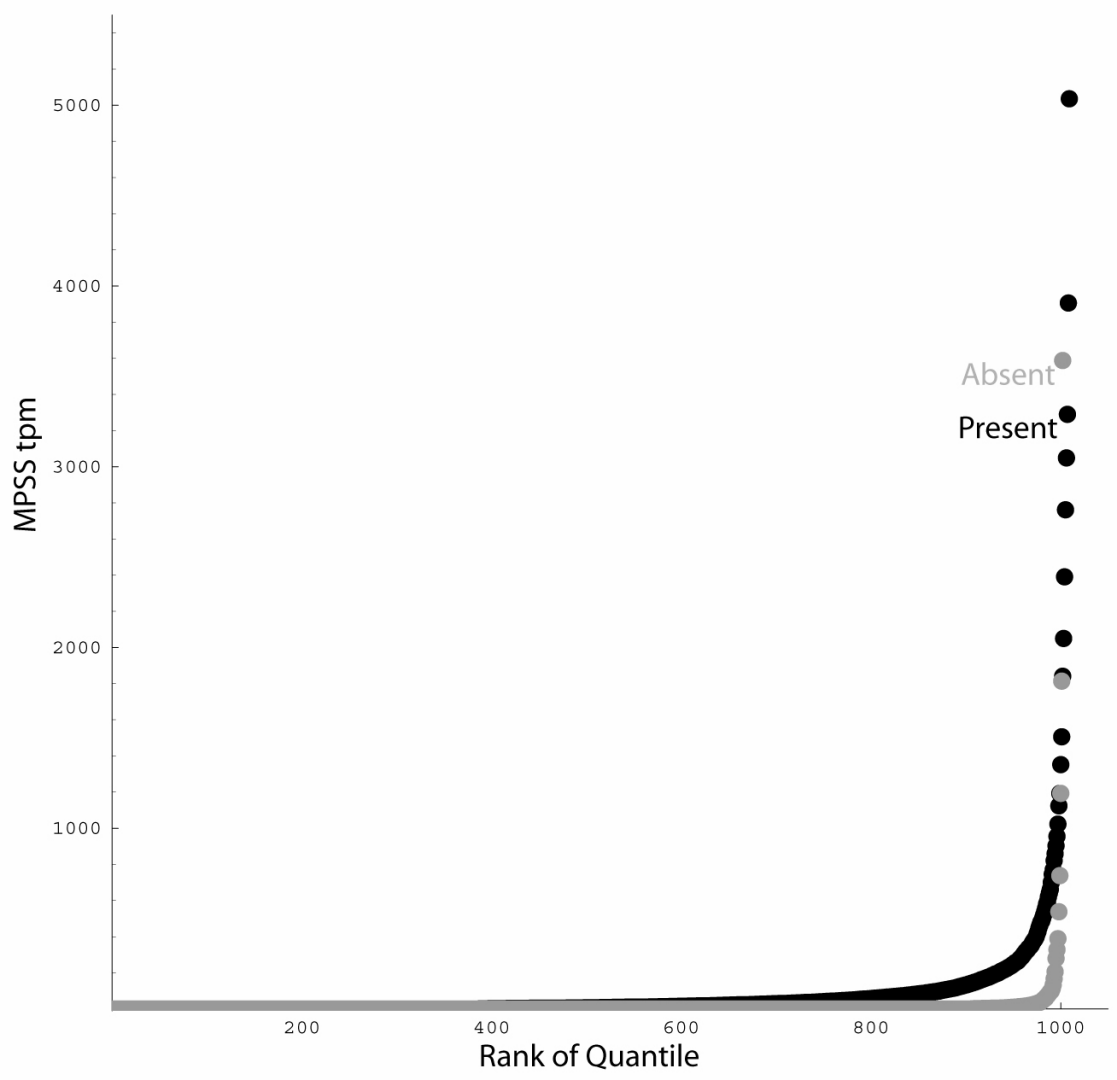
**Comparison of Affymetrix call with MPSS detection. **A rank-order plot of Affymetrix ProbeSet detection calls correlated with MPSS tpm for each GeneID is shown. The MPSS tpm associated with GeneID determined to be present (black) or absent (gray) were sorted by rank order, and then one thousand values were plotted, representing the 1/1000^th ^quantiles. Very few genes predicted to be absent by Affymetrix have a large MPSS tpm; a number of genes predicted to be present by Affymetrix have a low MPSS tpm.

The GCOS "detection P-value" is endorsed by Affymetrix as a tool for classifying transcript presence or absence. There are three possible values: (1) "present", for detection P-values < 0.04, (2) "absent", for P-values ≥ 0.06, and (3) "marginal" for intermediate P-values. To estimate the effect of using these default P-value cut-offs for declaring a gene present or absent, we correlated them for each GeneID to the tpm obtained by MPSS. We observed that GeneIDs with an "absent" detection call have a 91% chance of also having zero MPSS tpm, but also have a 2% chance of having a MPSS tpm of more than 30 (Fig. 3). GeneIDs with a "present" detection call have a 45% chance of also having an MPSS tpm greater than 10, but a 39% chance of having zero MPSS tpm.

### Detection of cell line-specific genes

**Figure 4:**
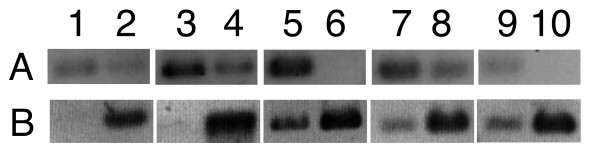
**RT-PCR verification of unique gene expression. **(A) C4-2 genes: Lane 1–2 *ARHE*, 3–4 *PHF11*, 5–6 *PLD1*, 7–8 *PRG-3*, and 9–10 *ACAS2L*. (B) LNCaP genes: Lane 1–2 *ETV1*, 3–4 *EPHA7*, 5–6 *FLJ12895*, 7–8 *MBNL2*, and 9–10 *PCDH11X*. Odd lanes are C4-2 and even lanes are LNCaP cDNA. Many genes determined to be unique to a cell line by Affymetrix and MPSS analysis were actually not when assayed by RT-PCR.

The Affymetrix and MPSS data was analyzed to determine which genes were "unique" to either cell line. For example, to be considered "unique" to C4-2 cells a gene was required to have an Affymetrix call of absent and not be detected by MPSS in LNCaP cells. Lists of the genes that are "unique" to C4-2 [see Additional file 3] and LNCaP [see Additional file 4] according to both Affymetrix and MPSS were compiled. C4-2 expressed 33 "unique" genes while LNCaP expressed 66 "unique" genes. We performed RT-PCR on all 99 "unique" genes to confirm that they were indeed unique or actually differentially expressed at levels not detected by these expression-profiling methods. Gene-specific primers were designed for all "unique" genes. The result for a subset of genes is presented in Fig. 4. From the C4-2 gene list *ARHE*, *PHF1*, *PLD1*, *PRG-3*, and *ACAS2L *were selected, and of these 5 genes, *PLD1 *and *ACAS2L *were confirmed to be uniquely expressed by C4-2; but *ARHE*, *PHF1*, and *PRG-3 *were not. The RT-PCR results suggest that *ARHE*, *PHF1*, and *PRG-3 *are likely differentially expressed and not unique. From the LNCaP gene list *PCDH11X*, *MBNL2*, *EPHA7*, *FLJ12895*, and *ETV1 *were selected, and of these, *ETV1 *and *EPHA7 *were confirmed to only be expressed by LNCaP cells, but *PCDH11X*, *MBNL2*, and *FLJ12895 *were not. Results for the other "unique" genes are compiled as the presence (+) or absence (-) of RT-PCR signal [see Additional files 3 and Additional file 4]. Of the 33 C4-2 genes *PLD1*, *ACAS2L*, *CA1*, *CA9*, *ITM2A*, *CARD14*, and *GPR54 *were verified to be uniquely expressed. Of the 66 LNCaP genes *ETV1*, *PAK1*, *EPHA7*, and *HELLS *were confirmed to be expressed only by LNCaP cells.

### MPSS "zeros"

**Figure 5:**
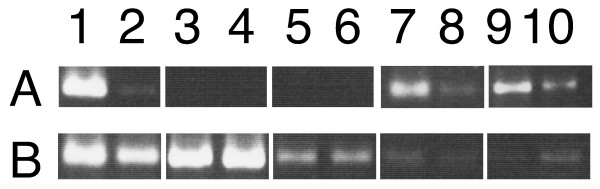
**RT-PCR verification of genes detected by Affymetrix analysis but not by MPSS. **All genes were determined to be expressed at 0 tpm by MPSS and detected present in C4-2 (A) or LNCaP (B) Affymetrix data. (A) Lane 1–2 *UGT8*, 3–4 *FOXQ1*, 5–6 *ZNF533*, 7–8 *GALC*, and 9–10 *FLJ23259*. (B) Lane 1–2 *ZNF625*, 3–4 *TBRG4*, 5–6 *TRPV6*, 7–8 *SLC4A11*, and 9–10 *LOC286097*. Odd lanes are C4-2 and even lanes are LNCaP cDNA. The expression of genes not detected by MPSS but detected by Affymetrix was verified with RT-PCR.

MPSS analysis reports zero tpm for some genes that are predicted to have an appropriate signature [24]. However, other methods of detection such as RT-PCR or Northern blot show that the gene is actually present in the RNA sample [9,26]. To confirm the concordance between an expression level of zero for a gene by MPSS and an Affymetrix signal for the same gene we performed RT-PCR on five genes expressed in C4-2 cells but not LNCaP and five genes expressed in LNCaP cells but not C4-2 as determined by Affymetrix (Fig. 5). All ten genes were not detected in C4-2 or LNCaP by MPSS. Of the C4-2 genes *UGT8*, *GALC*, and *FLJ23259 *were detected in both C4-2 and LNCaP although at very low levels in LNCaP. Two genes, *FOXQ1 *and *ZNF533 *were not detected in either C4-2 or LNCaP cells. Of the LNCaP genes *ZNF625*, *TBRG4*, and *TRPV6 *were detected in both C4-2 and LNCaP, and *SLC4A11 *was detected at very low levels in both. *LOC286097 *expression was only detected in LNCaP cells. These results suggest that the MPSS zero phenomenon is not a major problem for our study since the genes were otherwise detected by Affymetrix analysis.

### Expression of cell line-specific genes in prostate cancer metastases

**Figure 6:**
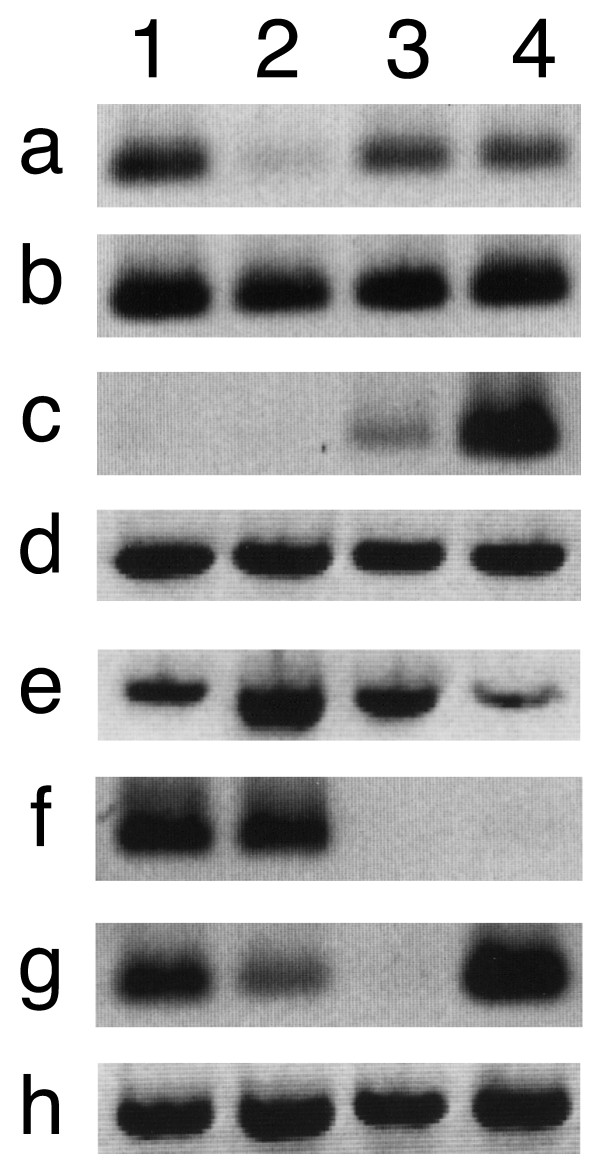
**Expression of C4-2 or LNCaP genes in prostate cancer. **Shown are the results of RT-PCR analysis of C4-2 (a-e) and LNCaP (f-h) genes in benign prostate (lane 1), primary tumor (lane 2), lymph node metastases (lane 3), and bone metastases (lane 4). The tested genes are: (a) *PRG-3*, (b) *PLD1*, (c) *CA1*, (d) *ACA2SL*, (e) *CARD14*, (f) *EPHA7*, (g) *ETV1*, and (h) *PAK1*. Genes unique to cancer cell lines displayed differential expression in prostate and prostate cancer metastases.

The LNCaP and C4-2 unique genes were assayed by RT-PCR for their expression in benign prostate, primary prostate cancer, lymph node metastasis, and bone metastasis (Fig. 6). The expression of each gene was determined in 3 different biological replicates and was consistent among the replicates; figure 6 data is representative of the replicates. The C4-2 genes *PLD1*, *PRG-3*, *ACAS2L *and *CARD14 *were expressed in all tissue types. *CARD14 *expression appeared to be greater in primary cancer and reduced in bone metastasis while that of *ACAS2L *and *PLD1 *appeared to be equivalent. *PRG-3 *appeared to be expressed at high levels in all samples except primary prostate cancer where the expression level appeared low. Expression of *CA1 *appeared to be high in bone metastasis and low in lymph node metastasis. The LNCaP gene *EPHA7 *was expressed in normal prostate and primary prostate cancer only. Expression of *ETV1 *was detected in all tissue types except lymph node metastasis. *PAK1 *was expressed in all tissue types. We also assayed the expression of the LNCaP gene *HELLS *and the C4-2 genes *CA9*, *ITM2A*, and *GPR54*. Since RT-PCR determined the expression of *HELLS*, *CA9*, *ITM2A*, and *GPR54 *were variable among the test samples, a representative expression profile was not included in figure 6.

## Discussion

The strengths of microarrays and MPSS appear somewhat complementary to one another. The degree to which microarray and MPSS data correlate is valuable information for researchers involved in gene expression studies. The two technologies could theoretically provide genome-wide coverage of a transcriptome. In practice, our data shows that Affymetrix or MPSS alone does not cover the transcriptome of LNCaP and C4-2 cell lines as evidenced by the detection of certain genes by one technology but not the other. Therefore, previous single-technique studies of LNCaP and C4-2 gene expression [27-29] have likely captured parts of their transcriptomes. Our merged Affymetrix and MPSS data have 11,010 genes for the LNCaP transcriptome and 10,667 genes for the C4-2 transcriptome; we believe that the numbers represent a reasonably complete profile of the genes that are expressed by these cells within the sensitivity range of the technologies. However, a comparison of the Affymetrix and MPSS data revealed a potentially surprising finding in that the expression of thousands of genes was not corroborated by the two technologies. In the LNCaP transcriptome, 28.9% of the genes were only detected by Affymetrix and 10.6% only by MPSS. In the C4-2 transcriptome, 38.6% of the genes were only detected by Affymetrix and 9.5% only by MPSS. Overall, we see that the Affymetrix signals are correlated with MPSS tpm. It is likely that some variability in this correlation comes from both detection processes. However, we note that at high tpm the signal strength tends to slow its increase with respect to tpm. The curve flattening suggests that ProbeSet signals may saturate for highly expressed transcripts, which has been previously observed by James *et al *[30]. Such saturation, however, does not pose a problem for an experimental design such as ours that focuses on the presence and absence of particular transcripts.

The GCOS "detection P-value" is recommended by Affymetrix to assess the presence or absence of a gene in an experiment. Our data shows that when the Affymetrix detection call is related to MPSS tpm value for genes called "absent" by GCOS greater than 90% of the genes also have a tpm of zero, which indicates that the technologies have a similar level of low-end sensitivity. However, when genes called "present" by GCOS are compared to MPSS tpm only 45% of genes also have a tpm greater than 10 and 39% of the genes have zero MPSS tpm. Many of these zeros are likely to be due to the failure of MPSS to measure certain splice forms of some GeneIDs, particularly those missing *Dpn*II sites. Given the low correlation of Affymetrix "present" calls and MPSS tpm the usefulness of the relationship as an absolute means to compare data sets is limited.

Due to the detection limits of Affymetrix and MPSS technologies further analysis of the genes "unique" to LNCaP and C4-2 cells was necessary. We used RT-PCR to determine the presence or absence of "unique" transcripts in the LNCaP and C4-2 cell lines. Of the 33 genes from the C4-2 "unique" list that we analyzed 21% were verified to be unique to C4-2 cells relative to LNCaP by RT-PCR. Of the 66 genes assayed from the LNCaP "unique" list 6% were verified to be unique to LNCaP cells by RT-PCR. In one case, our RT-PCR verification appears to validate MPSS signals as low as 1 tpm. The gene *PHLDA1 *had a C4-2 Affymetrix signal of 105 and was detected in both C4-2 and LNCaP RNA by RT-PCR [see Additional file 2]. An interesting aspect of the RT-PCR verification was the detection of many transcripts that were "absent" as determined by GCOS and had zero tpm. Qualitatively, it appears that the majority (89%) of the "absent" and zero tpm genes detected by RT-PCR in both cell lines are actually differentially expressed. Therefore, it may be more appropriate to interpret an Affymetrix "absent" call or an MPSS zero as a failure of the technology to detect the transcript and not its absence.

The biological reason for comparing LNCaP and C4-2 was to identify genes associated with cancer progression. C4-2 is a more malignant progeny of LNCaP produced through an *in vivo *process involving interaction between LNCaP and human bone stromal cells [31]. Unlike LNCaP, C4-2 has metastatic potential and is hormone insensitive. We postulated that C4-2 genes were likely to be found in advanced cancers; the strongest candidate was carbonic anhydrase 1 (*CA1*), expression of its transcript was restricted to metastases with a possible increase in bone. Our data suggests that *CA1 *expression is related to the progression of prostate cancer from tissue-localized disease where the gene is not expressed to metastasis where the gene is present. The expression data suggests that clones expressing *CA1 *are selected in bone metastasis. The expression pattern of the other tested genes was less notable in this regard. *PRG-3 *[32], an enzymatically inactive member of the recently described plasticity-related gene family of lipid phosphate phosphatases [33] is present in normal prostate but appears to be expressed at a lower level in primary tumor. Its expression pattern suggests that *PRG-3 *may be expressed in basal cells in normal glands as these cells are missing in tumor glands. Caspase recruitment domain family member 14 (*CARD14*), expression appeared to be elevated in primary cancer but reduced in lymph node and bone metastasis. *CARD14 *has been shown to interact with the apoptosis activator BCL10 and activate NF-κB [34]. The increased expression of *CARD14 *may facilitate the activation of NF-κB in prostate cancer. Ephrin receptor A7 (*EPHA7*) is a member of a large class of cell-cell communication receptor-ligand pairs, expression of which was detected in benign tissue and primary tumor but not in lymph node or bone metastasis. The expression of *EPHA7 *has been observed to be elevated in liver tumors, decreased in colon tumors, and unchanged in lung or kidney tumors [35]. It is interesting to note that increased expression of another ephrin receptor, *EPHA2*, has been demonstrated in prostatic intraepithelial neoplasia, and shown to be associated with neoplastic transformation [36]. Finally, ETS translocation variant 1 (*ETV1*), a member of the ETS transcription factor family, was expressed in normal and primary tumor, not in lymph node metastasis (although it is a LNCaP unique gene), and potentially elevated in bone metastasis. Greater expression of *ETV1 *may promote an aggressive phenotype of metastasis through its recently documented activation of human telomerase reverse transcriptase [37].

## Conclusion

The data we have presented represents the first direct comparison of Affymetrix gene expression profiling with MPSS. Given the methodological differences between the expression analysis platforms it is not surprising that the transcriptomes would differ, however, the degree to which the datasets diverge is surprising. The differences we have observed between Affymetrix and MPSS transcriptomes strongly suggest that a more complete transcriptome will be obtained when both technologies are employed. The use of multiple expression analysis platforms could be extended to other technologies such as cDNA microarrays, cDNA library sequencing, or SAGE and would likely further enhance the accuracy of the transcriptomes produced. In a practical application of multiple expression-platform profiling, our analysis of C4-2 and LNCaP prostate cancer cell lines has identified genes such as *CA1*, *CARD14*, and *EPHA7 *that may be involved in prostate cancer progression. In conclusion, the use of multiple transcriptome profiling methodologies will provide more complete datasets and may supply more reliable candidate genes for further investigation.

## Competing interests

The author(s) declare that they have no competing interests.

## Authors' contributions

AO co-designed the study, carried out Affymetrix experiments, performed RT-PCR experiments, analyzed data, and prepared the manuscript. JR analyzed Affymetrix and MPSS data with software he produced. JR also contributed to preparation of the manuscript. LW performed RT-PCR experiments. LE performed RT-PCR experiments. LT contributed to preparation of the manuscript. RV and LT contributed the human primary tissue cDNA samples used to examine expression patterns of candidate genes in prostate cancer progression. AL co-designed the study and contributed to the preparation of the manuscript.

## Pre-publication history

The pre-publication history for this paper can be accessed here:



## Supplementary Material

Additional File 1 **PCR primer sequences. **Nucleic acid sequences of PCR primer pairs for C4-2 specific, LNCaP specific, and MPSS zero genes. Click here for file

Additional File 2 **Overall Affymetrix and MPSS merge results. **List of GeneIDs detected by Affymetrix and MPSS expression profiling of C4-2 and LNCaP cell lines with corresponding signal or tpm data. Empty cells indicate absence of data. Columns contain the following data: GeneID (NCBI GeneID), X_P (Affymetrix present call 1 or absent call 0), X_S (Affymetrix raw signal), X_tpm (MPSS tpm for the gene), where X = cell line. Cell lines are labeled as follows: LN = LNCaP and C42 = C4-2. Click here for file

Additional File 3 **C4-2 specific genes. **Genes determined to be unique to C4-2 cells by Affymetrix signal and MPSS tpm. Gene expression was tested by RT-PCR, and PLD1, ACAS2L, CA1, ITM2A, CA9, CARD14, GPR54 were detected in C4-2 and not in LNCaP. Columns contain the following data: GeneID (NCBI GeneID), X_S (Affymetrix raw signal), X_tpm (MPSS tpm), X_RT (RT-PCR result, positive + or negative -), where X = cell line. Cell lines are labeled as follows: LN = LNCaP and C42 = C4-2. Click here for file

Additional File 4 **LNCaP specific genes. **Genes determined to be unique to LNCaP cells by Affymetrix signal and MPSS tpm. Gene expression was tested by RT-PCR, and ETV1, PAK1, EPHA7, HELLS, were detected in LNCaP and not in C4-2. Columns contain the following data: GeneID (NCBI GeneID), X_S (Affymetrix raw signal), X_tpm (MPSS tpm), X_RT (RT-PCR result, positive + or negative -), where X = cell line. Cell lines are labeled as follows: LN = LNCaP and C42 = C4-2. Click here for file
